# Supplementary material for: Extracellular vesicles from diverse fungal pathogens induce species-specific and endocytosis-dependent immunomodulation
Source: bioRxiv. 2025 Jan 3:2025.01.03.631181. Preprint. [Version 1] doi: 10.1101/2025.01.03.631181 (PMC11722428; doi:10.1101/2025.01.03.631181)

bioRxiv preprint doi: https://doi.org/10.1101/2025.01.03.631181; this version posted January 3, 2025. The copyright holder for this preprint (which was not certified by peer review) is the author/funder, who has granted bioRxiv a license to display the preprint in perpetuity. It is made available under aCC-BY 4.0 International license.

Supplemental Figures

Fig S1. Isolation of fungal EVs. Isolation procedures of EVs from *C. albicans*, *S. cerevisiae*, *C. neoformans,* and *A. fumigatus* cultures.

Fig S2. Immunomodulatory properties of fungal EVs. A, Heat map of selected cytokines secreted by WT and *Sting*$^{-/-}$ macrophages when stimulated by PBS, cGAMP, *Ca* EVs, *Sc* EVs, *Cn* EVs, and *Af* EVs (EVs added at $1x10^{10}$ EVs/mL). **b,** Immunoblots of viperin and actin in WT and *Sting*$^{-/-}$ macrophages stimulated by PBS, 2.5µg cGAMP, *Ca* EVs, or *Sc* EVs (EVs added at $1x10^{10}$ EVs/mL). **c,** Immunoblot of viperin and actin in WT and *Sting*$^{-/-}$ macrophages stimulated by PBS, 2.5µg cGAMP, *Cn* EVs, or *Af* EVs (EVs added at $1x10^{10}$ EVs/mL).

Figure S1

bioRxiv preprint doi: https://doi.org/10.1101/2025.01.03.631181; this version posted January 3, 2025. The copyright holder for this preprint (which was not certified by peer review) is the author/funder, who has granted bioRxiv a license to display the preprint in perpetuity. It is made available under aCC-BY 4.0 International license.

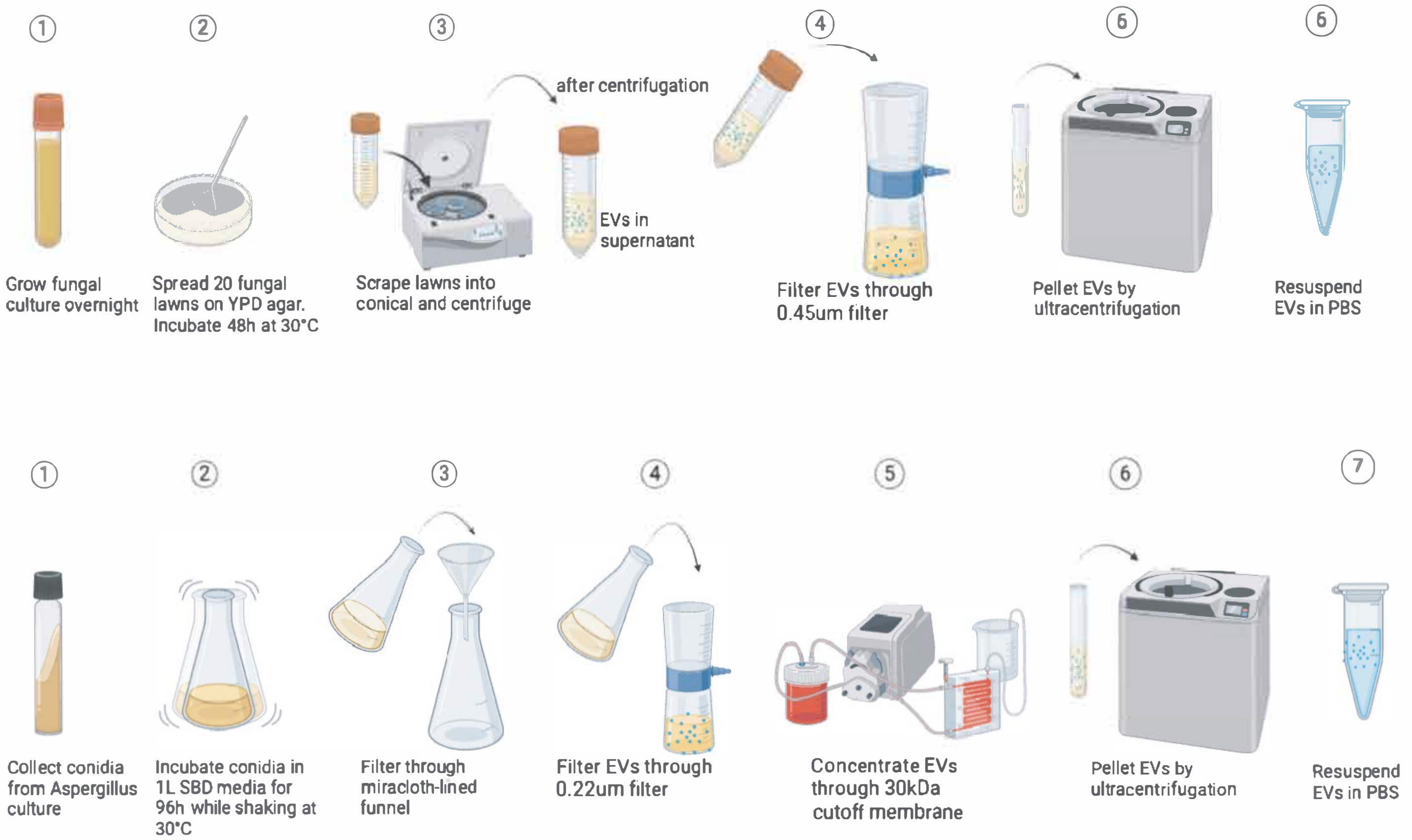

bioRxiv preprint doi: https://doi.org/10.1101/2025.01.03.631181; this version posted January 3, 2025. The copyright holder for this preprint (which was not certified by peer review) is the author/funder, who has granted bioRxiv a license to display the preprint in perpetuity. It is made available under aCC-BY 4.0 International license.

Figure S2

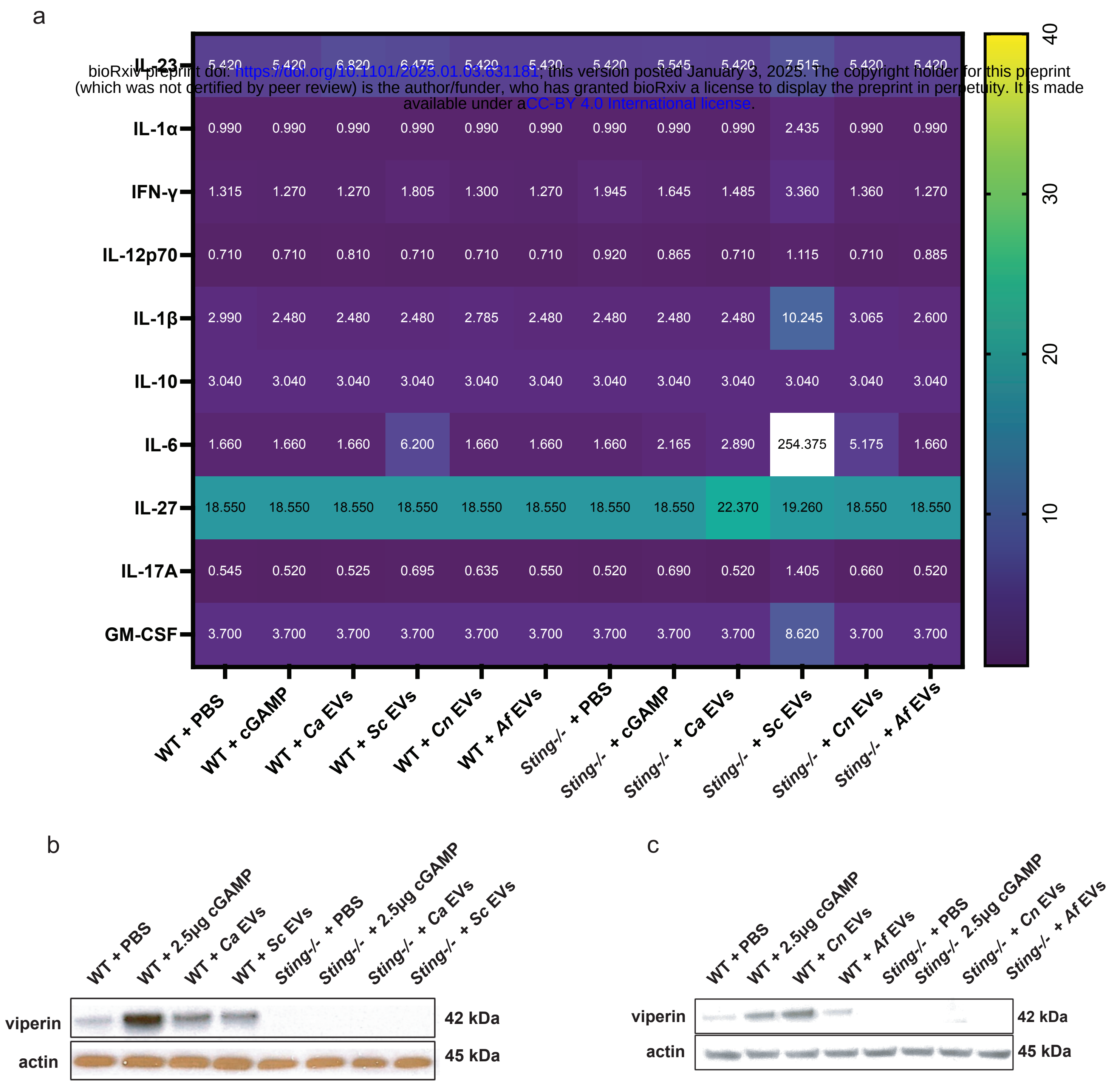

Supplement: 1 [file NIHPP2025.01.03.631181v1-supplement-1.pdf]
